# Donor artery stenosis interactions with diastolic blood pressure on coronary collateral flow in type 2 diabetic patients with chronic total occlusion

**DOI:** 10.1186/s12933-018-0724-x

**Published:** 2018-06-01

**Authors:** Ying Shen, Zhen Kun Yang, Jian Hu, Xiao Qun Wang, Yang Dai, Su Zhang, Rui Yan Zhang, Lin Lu, Feng Hua Ding, Wei Feng Shen

**Affiliations:** 1 Department of Cardiology, Rui Jin Hospital, Shanghai Jiao Tong University School of Medicine, 197 Rui Jin Road II, Shanghai, 200025 People’s Republic of China; 2 Institute of Cardiovascular Diseases, Shanghai Jiao Tong University School of Medicine, 197 Rui Jin Road II, Shanghai, 200025 People’s Republic of China; 3 College of Biomedical Engineering, Jiao Tong University, Shanghai, 200031 People’s Republic of China

**Keywords:** Blood pressure, Collateral circulation, Diabetes, Coronary artery disease, Chronic total occlusion

## Abstract

**Background:**

We investigated whether and to what extent stenosis of predominant collateral donor artery (PCDA) affects coronary collateral flow in relation to blood pressure (BP) in type 2 diabetic patients with chronic total occlusion (CTO).

**Methods:**

Collateral flow index (CFI) as derived from intracoronary pressure distal to occluded segment and mean aortic pressure in 220 type 2 diabetic patients and 220 propensity score matched non-diabetic controls undergoing percutaneous coronary intervention for CTO. The severity of PCDA stenosis was graded according to lumen diameter narrowing.

**Results:**

CFI decreased stepwise from mild to severe stenosis of the PCDA and was lower in diabetic patients with moderate or severe PCDA stenosis than in non-diabetic controls (0.36 ± 0.10 vs. 0.45 ± 0.08, P < 0.001; 0.29 ± 0.09 vs. 0.35 ± 0.08, P = 0.008). When the PCDA was mildly stenotic, CFI increased initially along with a reduction in diastolic BP, and decreased when diastolic BP was below 60 mmHg in diabetic patients (0.38 ± 0.16 vs. 0.57 ± 0.09, P < 0.001). In the presence of moderate PCDA stenosis, diabetic patients had significantly lower CFI compared to non-diabetic controls, with a relative reduction of 19.8% at diastolic BP 70–79 mmHg, 28.2% at 60–69 mmHg and 38.2% below 60 mmHg (all P < 0.05). A severe PCDA stenosis resulted in a more pronounced decrease in CFI, with a relative reduction of 37.3% for diabetics compared to non-diabetics when diastolic BP was below 60 mmHg (P = 0.050).

**Conclusions:**

In the setting of CTO, donor artery stenosis confers greater risk for reduced coronary collateral flow when diastolic BP is decreased. Even a moderate stenosis in the PCDA may be associated with lower collateral flow as diastolic BP decreases below 80 mmHg in type 2 diabetic than in non-diabetic patients.

## Background

In patients with stable coronary artery disease, a gradual development of complete coronary obstruction may lead to a sufficient compensation of blood supply via collateral circulation to prevent myocardial damage from ischemic insults [1]. Protection of the jeopardized myocardium by coronary collaterals is clinical relevant, as presence of well-formed collaterals has been associated with reductions in the occurrence and transmural extent of myocardial infarction, and with increased survival [2]. The mechanism of collateral vessel growth is complex in situations where atherosclerosis affects large conductance arteries [3], and even become more complicated by the presence of diabetes mellitus in which multiple biochemical and cellular components are involved [4]. Nevertheless, arteriogenesis with vessel outward remodeling is prevail, and weights much more than angiogenesis because it reduces dramatically collateral resistance to a negligible extent and enables delivery of blood flow to the region at risk [5, 6]. Among numerous factors which could influence coronary collateral flow [4, 7–10], blood pressure (BP), especially diastolic BP, generates the distal pressure within the occluded segment of the coronary artery, which constitutes a physical stimulus for arteriogenesis and promotes collateral formation [11]. Presence of a chronic total occlusion (CTO) is frequently associated with multi-vessel coronary disease and has been considered as a prerequisite for spontaneous collateral recruitment [7, 12]. Collaterals develop due to the pressure gradient from donor to recipient being greater than that of the recipient (often a CTO). Obviously, myocardium distal to the occlusion is almost entirely perfused by retrograde collateral branches from another epicardial coronary artery (i.e., predominant collateral donor artery [PCDA]), and successful recanalization of a chronically occluded lesion has often led to a rapid reduction of pressure-derived recruitable collateral function and an increase in fractional flow reserve of the PCDA [12]. This suggests a potential interaction between coronary collateral flow and donor artery physiology in the setting of chronic coronary total occlusion. However, the effect of donor artery stenosis on coronary collateral flow in relation to BP for patients with diabetes remains unknown, which partly reflects the heterogeneity of study population and semi-quantitative angiographic assessment of coronary collateral circulation in most previous cohort studies [8–10]. In this study, we investigated whether or to what extent combined BP (particularly diastolic BP) and stenosis of the PCDA affects coronary collateral flow in type 2 diabetic and non-diabetic patients with chronic coronary total occlusion. We examined the equilibrium of collateral supply at the time of advancing a microcatheter distal to the occlusion before antegrade flow is re-established. Collateral flow index (CFI) was derived from intracoronary distal occluded pressure and central aortic pressure taking into account of central venous pressure [7, 13, 14], which has been considered as the most accurate diagnostic tool to assess the capacity of coronary collateral circulation as it correlates closely with clinical signs of myocardial ischemia [15, 16]. For avoiding potential confounding factors, each type 2 diabetic patient was matched to a non-diabetic control for age, sex and risk factors for coronary artery disease.

## Methods

### Study population

**Fig. 1:**
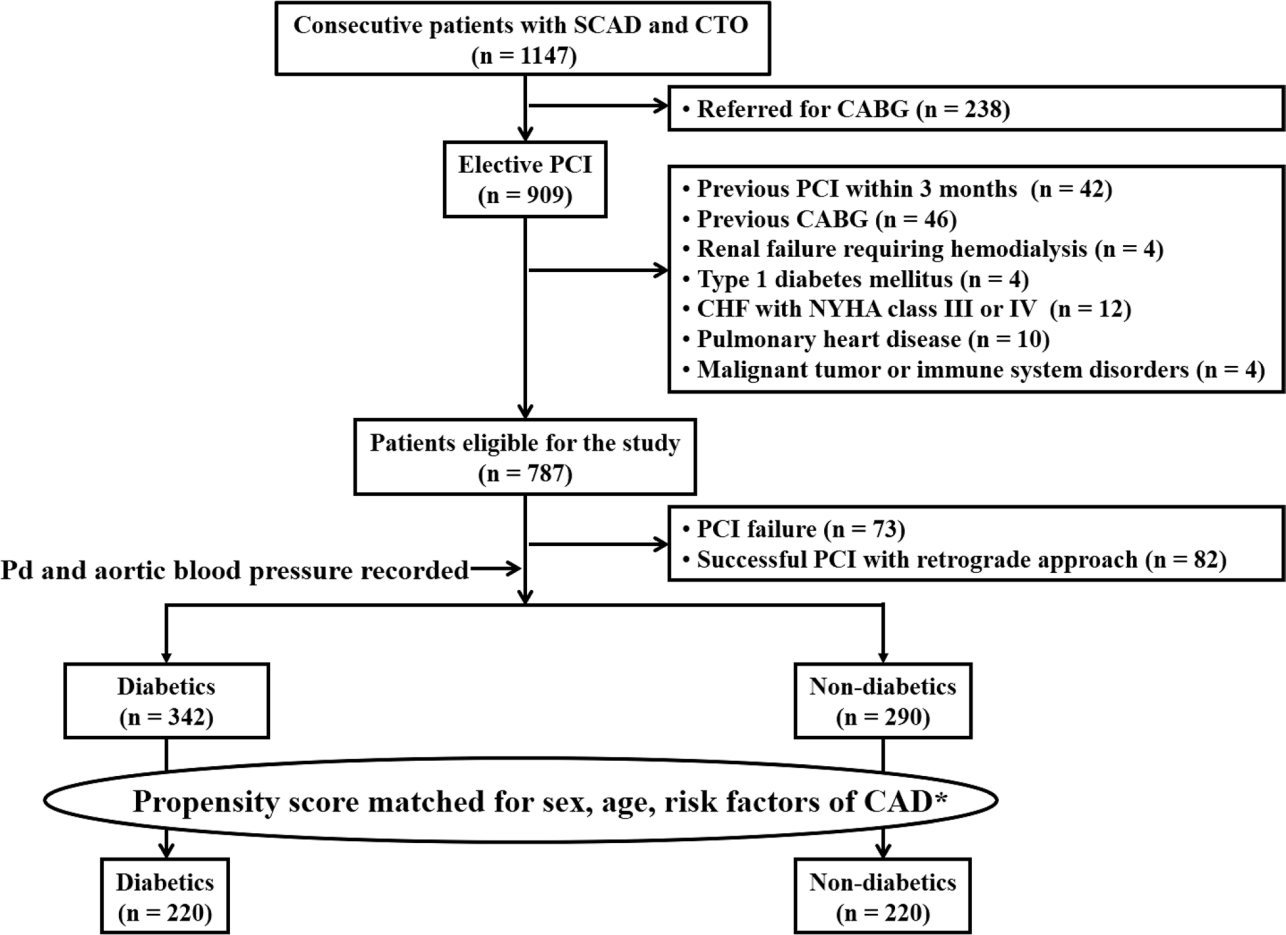
Flowchart of patient enrollment. *CABG* coronary artery bypass grafting, *SCAD* stable coronary artery disease, *CHF* chronic heart failure, *PCI* percutaneous coronary intervention, *Pd* mean intracoronary pressure distal to an occluded segment. *Risk factors for CAD indicate smoking, hypertension and dyslipidemia

In total, 1147 consecutive patients with stable coronary artery disease and CTO (> 3 months) of at least one major epicardial coronary artery between October 2010 and December 2016 were screened from the database of Shanghai Rui Jin Hospital PCI Outcome Program [17, 18]. We excluded 238 patients who were referred for coronary artery bypass grafting (CABG). In the remaining 909 patients undergoing elective PCI, we further excluded those who had a history of PCI within 3 months (n = 42) or CABG (n = 46), renal failure requiring hemodialysis (n = 4), type 1 diabetes (n = 4), chronic heart failure with NYHA class III or IV (n = 12), pulmonary heart disease (n = 10) and malignant tumor or immune system disorders (n = 4). We also excluded those who had failed PCI for CTO mostly due to inability of guide wire to cross the occluded segment (n = 73) and those who underwent PCI via a retrograde approach (n = 82). To reduce the selection bias, we then performed a propensity score matching analysis, resulting in a total number of 440 patients (220 type 2 diabetics and 220 non-diabetics) into the final analyses (Fig. 1).

The duration of CTO was estimated based on information obtained from a previous angiogram, a history of myocardial infarction in the target vessel territory, or the first onset of an abrupt worsening of existing angina. The diagnosis of type 2 diabetes was made according to the criteria of the American diabetes association, including glycosylated hemoglobin (HbA1c) ≥ 6.5%, fasting plasma glucose concentration ≥ 7.0 mmol/L, 2-h postprandial glucose concentration ≥ 11.1 mmol/L, or a random plasma glucose ≥ 11.1 mmol/L in a patient with classic symptoms of hyperglycemia or hyperglycemic crisis [19]. Hypertension was defined as systolic BP ≥ 140 mmHg and/or diastolic BP ≥ 90 mmHg, or use of anti-hypertensive agents for controlling BP [20]. Dyslipidemia was defined according to the Third Report of The National Cholesterol Education Program (NCEP) [21]. Stable angina was diagnosed according to the criteria recommended by the American College of Cardiology/American Heart Association [22].

### Biochemical investigation

We obtained blood samples at the day of angiography in all patients after an overnight fasting. Assessment of serum levels of creatinine, blood urea nitrogen, uric acid, lipid profiles, glucose, and glycosylated hemoglobin (HbA1c), was made with standard laboratory techniques. Glomerular filtration rate (GFR) was estimated using the chronic kidney disease epidemiology collaboration (CKD-EPI) equation: GFR_EPI_ (mL/min/1.73 m^2^) = 141 × min (creatinine/k, 1)^α^ × max (creatinine/k, 1)^−1.209^ × 0.993^age^ × 1.018 [if female], where k is 0.7 for females and 0.9 for males, α is − 0.329 for females and − 0.411 for males, min indicates the minimum of creatinine/k or 1, and max indicates the maximum of creatinine/k or 1 [23]. Serum high-sensitivity C-reactive protein (hsCRP) level was assayed by ELISA (Biocheck Laboratories, Toledo, OH, USA).

### Angiography and analysis

Coronary angiography was performed via femoral or radial access with 6Fr diagnostic catheters. Quantitative angiographic assessment was done independently by two blinded interventional cardiologists according to lesion classification scheme of the American College of Cardiology/American Heart Association [24]. The PCDA was defined as a contra-lateral vessel making the largest collateral contribution [25], and maximal diameter stenosis of the PCDA was classified as mild: < 50%, moderate: 50–70% or severe: > 70% (GE Centricity VA 1000; GE Healthcare).

### Intracoronary pressure and collateral flow measurement

Elective PCI for CTO was performed through an antegrade approach in all patients. After crossing the occluded segment with various types of guide wire, a microcatheter was advanced distal to the occlusion. Sometimes, a low-profile balloon catheter was passed initially, and then was exchanged for a microcatheter. Meticulous care was taken to ensure complete obstruction of the coronary artery by lack of contrast passage during proximal contrast injection through the guiding catheter while the microcatheter was in place. Intracoronary pressure distal to an occluded segment measured through a microcatheter (Finecross, Terumo Co, Japan) and central aortic pressure determined from a 6Fr guiding catheter were simultaneously recorded using a fluid-filled manometer system (Mac-Lab Hemodynamic Recording System, GE Healthcare, USA). The transducer was kept at the level of mid axillary line, and zero calibrated to atmosphere before measurement [17]. Despite a phasic waveform for distal coronary pressure, significant damping of high frequency pressure components by the small lumen of microcatheter exhibited, thus only the mean distal occluded pressure was used. For measurement of central aortic pressure, the guiding catheter was kept away from the coronary orifice. The pressure-derived coronary collateral flow index (CFI) was calculated as the ratio of (Pd − CVP)/(Pa − CVP), where Pd is mean distal occluded pressure, Pa is mean central aortic pressure; and CVP is the central venous pressure, which was substituted by 5 mmHg, as all patients had no symptoms and signs of heart failure [7, 14, 15].

We have validated the accuracy of intracoronary pressure-derived collateral flow measurement using a microcatheter against the standard pressure wire technique (PressureWire™ Certus, St. Jude Medical, St. Paul, Minnesota) with RadiAnalyzer™ Xpress System (St. Jude Medical, St. Paul, Minnesota). Bland–Altman analysis revealed that in 40 consecutive patients, Pd and CFI determined with a microcatheter correlated significantly with those by a pressure wire (r = 0.751 and r = 0.679, both P < 0.001), with an absolute difference in Pd and CFI between the two techniques of 1.68 mmHg (95% CI − 1.42 to 4.77, P = 0.280) and 0.019 (95% CI − 0.02 to 0.053, P = 0.279), respectively.

### Statistical analysis

For patient selection, a propensity score matching analysis was performed in advance with a match tolerance of 0.02 and a ratio of 1:1 for diabetic and non-diabetic patients using a logistic regression model with age, sex and risk factors for coronary artery disease (smoking and history of hypertension or dyslipidemia). Continuous variables are presented as mean ± standard deviation (SD), and categorical data are summarized as frequencies (percentages). For categorical clinical variables, differences between groups were evaluated with the Chi square test. For continuous variables, the existence of a normal distribution was evaluated with the Kolmogorov–Smirnov test, and differences among groups were analyzed by one-way analysis of variance (ANOVA) followed by post hoc analysis with the Fisher’s least significant difference (LSD) test. Pearson’s or Spearman’s correlation analysis was run to determine the relationship between CFI and Pd or different levels of aortic BP. Multivariate linear regressions were used to explore the independent determinants for CFI, and the covariates chosen to enter the multivariate analysis model included diabetes and aortic systolic, diastolic and mean BP, respectively, as well as gender, age, body mass index (BMI), risk factors for coronary artery disease (history of hypertension and dyslipidemia and smoking), history of myocardial infarction, severity of coronary artery disease, GFR, total to high-density lipoprotein (HDL) cholesterol ratio, log-transferred hsCRP and left ventricular ejection fraction. All analyses used 2-sided tests with an overall significance level of alpha = 0.05, and were performed with the SPSS 20.0 for Windows (SPSS, Inc., Chicago, IL, USA).

## Results

### Baseline characteristics

Mild, moderate and severe stenosis of the PCDA was observed in 99, 75 and 46 diabetic patients and 132, 60 and 28 non-diabetic patients, respectively. For diabetic and non-diabetic patients, age, proportion of male gender, dyslipidemia and prior myocardial infarction, and serum levels of creatinine and hsCRP increased whereas GFR, left ventricular ejection fraction and proportion of hypertension decreased stepwise from mild to severe stenosis of the PDCA (all P < 0.05). Multivessel coronary disease was more prevalent in patients with moderate or severe PDCA stenosis. Medications were similar irrespective of stenosis severity of the PDCA (Table 1).

**Table 1:** Baseline characteristics in patients with stable coronary artery disease and chronic total occlusion with mild, intermediate and severe stenosis of donor artery

Variables	Diabetes (n = 220)				Non-diabetes (n = 220)			
	Mild (n = 99)	Moderate (n = 75)	Severe (n = 46)	P value	Mild (n = 132)	Moderate (n = 60)	Severe (n = 28)	P value
Male, n (%)	94 (94.9)	59 (78.7)	24 (52.2)	< 0.001	127 (96.2)	40 (66.7)	14 (50.0)	< 0.001
Age, years	57.9 ± 11.7	67.9 ± 10.7	73.7 ± 9.3	< 0.001	61.3 ± 12.1	68.0 ± 9.3	74.6 ± 12.4	< 0.001
Body mass index, kg/m^2^	25.4 ± 2.5	25.5 ± 2.6	25.9 ± 2.1	0.605	25.0 ± 2.5	25.0 ± 1.9	25.7 ± 2.4	0.294
Hypertension, n (%)	102 (77.3)	37 (61.7)	7 (25.0)	< 0.001	91 (91.9)	42 (56.0)	11 (23.9)	< 0.001
History of dyslipidemia, n (%)	23 (17.4)	25 (41.7)	14 (50.0)	< 0.001	2 (2.0)	20 (26.7)	26 (56.5)	< 0.001
Smoking, n (%)	27 (27.3)	26 (34.7)	21 (45.7)	0.090	43 (32.6)	18 (30.0)	15 (53.6)	0.072
History of MI, n (%)	19 (19.2)	16 (21.3)	19 (41.3)	0.012	19 (14.4)	12 (20.0)	10 (35.7)	0.030
Severity of CAD, n (%)				< 0.001				< 0.001
1-vessel	39 (39.4)	2 (2.7)	0 (0.0)	< 0.001	51 (38.6)	5 (8.3)	5 (17.9)	< 0.001
2-vessel	35 (35.4)	21 (28.0)	17 (37.0)	0.493	55 (41.7)	17 (28.3)	7 (25.0)	0.089
3-vessel	25 (25.2)	52 (69.3)	29 (63.0)	< 0.001	26 (19.7)	38 (63.3)	16 (57.1)	< 0.001
Multivessel disease	60 (60.6)	73 (97.3)	46 (100.0)	< 0.001	81 (61.4)	55 (91.7)	23 (82.1)	< 0.001
Stenosis of PCDA, %	37.6 ± 8.5	58.9 ± 5.3	83.4 ± 8.5	< 0.001	33.5 ± 10.5	58.9 ± 5.4	82.6 ± 8.4	< 0.001
Fast blood glucose, mmol/L	6.3 ± 1.9	6.9 ± 2.2	6.5 ± 2.7	0.214	5.0 ± 0.7	4.7 ± 0.7	5.3 ± 0.6	< 0.001
HbA1c, %	7.2 ± 1.2	7.4 ± 1.5	7.1 ± 1.3	0.555	5.7 ± 0.4	5.7 ± 0.4	5.8 ± 0.4	0.980
Triglyceride, mmol/L	1.55 ± 0.72	1.62 ± 0.63	1.68 ± 0.81	0.538	1.42 ± 0.71	1.45 ± 0.83	1.60 ± 0.71	0.521
Total cholesterol, mmol/L	3.75 ± 1.04	4.11 ± 1.12	4.29 ± 1.04	0.009	4.11 ± 1.34	3.89 ± 1.45	4.08 ± 0.88	0.571
HDL cholesterol, mmol/L	0.94 ± 0.20	0.99 ± 0.28	0.99 ± 0.16	0.312	1.01 ± 0.27	0.96 ± 0.19	1.00 ± 0.34	0.469
LDL cholesterol, mmol/L	2.23 ± 0.90	2.47 ± 0.93	2.71 ± 0.87	0.010	2.49 ± 1.05	2.45 ± 1.11	2.40 ± 0.84	0.891
Serum creatinine, μmol/L	85 ± 21	90 ± 30	110 ± 32	< 0.001	77 ± 14	94 ± 27	102 ± 29	< 0.001
Uric acid, μmol/L	322 ± 79	315 ± 61	331 ± 76	0.515	337 ± 72	319 ± 96	339 ± 69	0.309
GFR, mL/min/1.73 m^2^	86.8 ± 18.5	75.5 ± 24.9	55.9 ± 20.6	< 0.001	90.3 ± 15.8	70.7 ± 20.2	59.6 ± 21.8	< 0.001
hsCRP, mg/L	1.58 (0.66–3.32)	2.46 (1.21–7.55)	4.23 (1.44–17.15)	0.031	0.97 (0.25–3.13)	1.20 (0.27–10.35)	2.66 (0.42–25.82)	< 0.001
LVEF, %	60.5 ± 8.3	60.4 ± 9.3	54.7 ± 10.7	0.001	62.3 ± 9.0	61.3 ± 9.5	57.2 ± 12.8	0.042
Medication, n (%)								
ACE inhibitors/ARBs	53 (53.5)	42 (56.0)	26 (56.5)	0.924	50 (37.9)	20 (33.3)	11 (39.3)	0.798
β blockers	41 (41.4)	30 (40.0)	20 (43.5)	0.931	51 (38.6)	25 (41.7)	15 (53.6)	0.345
Calcium channel blockers	30 (30.3)	23 (30.7)	12 (26.1)	0.845	41 (31.1)	13 (21.7)	8 (28.6)	0.406
Nitrates	57 (57.6)	41 (54.7)	24 (52.2)	0.819	59 (44.7)	32 (53.3)	16 (57.1)	0.339
Statins	69 (69.7)	59 (78.7)	36 (78.3)	0.328	77 (58.3)	33 (55.0)	12 (42.9)	0.325
Anti-diabetic therapy	58 (58.6)	60 (80.0)	40 (87.0)	< 0.001	0 (0.0)	0 (0.0)	0 (0.0)	–
Aortic blood pressure, mmHg								
Systolic	150.2 ± 26.2	147.3 ± 23.4	141.2 ± 24.8	0.130	150.2 ± 29.2	147.0 ± 28.2	141.0 ± 17.9	0.260
Diastolic	77.1 ± 9.8	74.9 ± 9.4	73.5 ± 10.8	0.093	78.1 ± 13.2	75.9 ± 9.7	75.6 ± 11.5	0.388
Mean	101.5 ± 13.2	99.0 ± 11.8	96.1 ± 13.5	0.057	101.2 ± 14.7	101 ± 12.9	97.4 ± 7.7	0.407
Pd, mmHg	52.1 ± 9.4	39.6 ± 11.0	32.5 ± 10.2	< 0.001	53.6 ± 7.1	48.3 ± 8.6	35.6 ± 7.8	< 0.001
Collateral flow index	0.49 ± 0.09	0.36 ± 0.10	0.29 ± 0.09	< 0.001	0.51 ± 0.08	0.45 ± 0.08	0.35 ± 0.08	< 0.001

Data are mean ± SD or median (25th–75th percentiles) or number (%)

*ACE* angiotensin converting enzyme, *ARB* angiotensin receptor blocker, *CAD* coronary artery disease, *GFR* glomerular filtration rate, *HbA1c* glycosylated hemoglobin A1c, *HDL* high-density lipoprotein, *hsCRP* high-sensitivity C reactive protein, *LDL* low-density lipoprotein, *LVEF* left ventricular ejection fraction, *MI* myocardial infarction, *PCDA* predominant collateral donor artery, *Pd* mean pressure distal to occlusion

### Collateral flow and BP

**Fig. 2:**
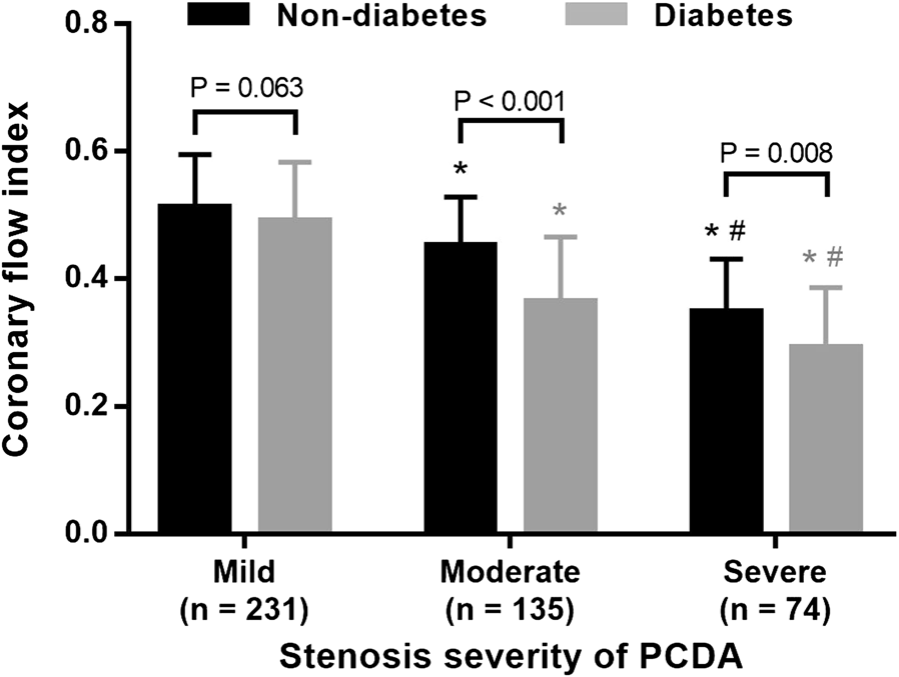
Comparison of CFI between diabetic and non-diabetic patients with mild, moderate or severe stenosis of PCDA group. *P < 0.001 vs. mild; #P < 0.001 vs. moderate

Despite similar aortic systolic, diastolic and mean BP (Table 1), CFI decreased stepwise from mild to severe stenosis of the PDCA in diabetic and non-diabetic patients (both P < 0.001), and were significantly lower in diabetic patients than in non-diabetic controls with moderate (0.36 ± 0.10 vs. 0.45 ± 0.08, P < 0.001) or severe (0.29 ± 0.09 vs. 0.35 ± 0.08, P = 0.008) PCDA stenosis. Furthermore, there was no difference in CFI between diabetic patients with moderate PDCA stenosis and non-diabetic controls with severe PCDA stenosis (P = 0.421) (Fig. 2).

### Effect of medical therapy

Treatments with β-blockers and nitrates were associated with higher CFI in diabetic (0.42 ± 0.12 vs. 0.38 ± 0.13, P = 0.026; 0.42 ± 0.12 vs. 0.39 ± 0.12, P = 0.033) and non-diabetic (0.49 ± 0.09 vs. 0.46 ± 0.10, P = 0.023; 0.49 ± 0.10 vs. 0.46 ± 0.10, P = 0.019) patients whereas other medications such as angiotensin-converting enzyme inhibitors or angiotensin receptor blockers, calcium channel blockers, statins, oral hypoglycemic agents and insulin did not significantly affect CFI in both groups (all P > 0.05).

### Multivariable analysis

Multivariable linear regression models with systolic (per 20 mmHg), diastolic (per 10 mmHg) and mean (per 10 mmHg) BP as well as potential confounding variables such as gender, age, body mass index, history of hypertension and dyslipidemia, smoking, prior myocardial infarction, severity of coronary artery disease, GFR, total-to-HDL cholesterol ratio, log-transferred hsCRP and left ventricular ejection fraction, revealed that after multiple adjustments, CFI correlated negatively with systolic, diastolic, and mean BP in diabetic and non-diabetic patients with mild PCDA stenosis (P ≤ 0.001), was inversely related to diastolic BP in non-diabetic patients with moderate PCDA stenosis (P = 0.011), but correlated positively with diastolic BP in diabetic patients with severe PCDA stenosis (P = 0.001).

**Fig. 3:**
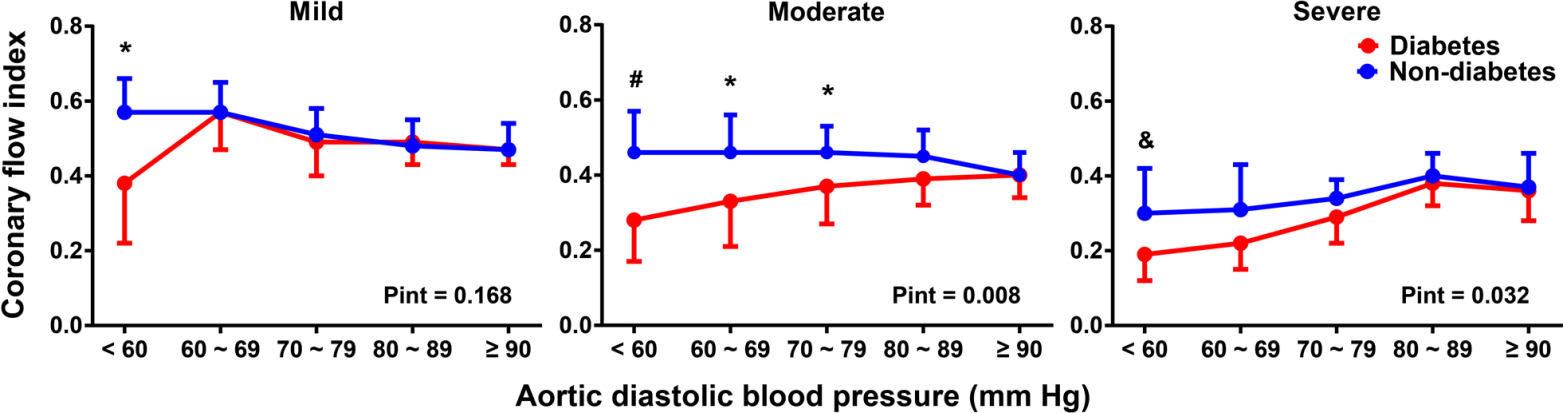
Correlation between CFI and aortic BP in diabetic (red) and non-diabetic (blue) patients with mild (left), moderate (middle) or severe (right) stenosis of the PCDA, respectively. *P < 0.001, #P < 0.01, and P < 0.05 vs. diabetics

Further analysis showed an interaction between diabetes and diastolic BP in patients with moderate (P interaction = 0.008) and severe (P interaction = 0.032) stenosis of the PCDA (Table 2). When the PCDA was mildly stenotic, CFI was gradually increased along with a reduction in aortic diastolic BP, but it was decreased when diastolic BP was below 60 mmHg in diabetic patients, with a relative reduction of 32.1% compared with non-diabetic controls (0.38 ± 0.16 vs. 0.57 ± 0.09, P < 0.001). In the presence of moderate PCDA stenosis, with decreasing diastolic BP, the difference of CFI between diabetic and non-diabetic patients was gradually increased. When diastolic BP was below 80 mmHg, diabetic patients had a significantly lower CFI compared to non-diabetic controls, with a relative reduction of 19.8% at diastolic BP 70–79 mmHg (0.37 ± 0.10 vs. 0.46 ± 0.07, P < 0.001), 28.2% at 60–69 mmHg (0.33 ± 0.12 vs. 0.46 ± 0.10, P < 0.001) and 38.2% below 60 mmHg (0.28 ± 0.11 vs. 0.46 ± 0.11, P = 0.002), respectively. A severe stenotic lesion in the PCDA led to more pronounced decrease in CFI, with a relative reduction of 37.3% for diabetic patients compared to non-diabetic controls when diastolic BP was below 60 mmHg (0.19 ± 0.07 vs. 0.30 ± 0.12, P = 0.050) (Fig. 3).

**Table 2:** Aortic blood pressure in relation to coronary flow index in diabetic and non-diabetic patients according to stenosis of PCDA

Aortic BP	Mild					Moderate					Severe				
	Diabetes (n = 99)		Non-diabetes (n = 132)		Pint	Diabetes (n = 75)		Non-diabetes (n = 60)		Pint	Diabetes (n = 46)		Non-diabetes (n = 28)		Pint
	β ± SE	P	β ± SE	P		β ± SE	P	β ± SE	P		β ± SE	P	β ± SE	P	
Systolic	− 0.284 ± 0.073	< 0.001	− 0.352 ± 0.070	< 0.001	0.325	− 0.013 ± 0.062	0.840	0.065 ± 0.158	0.682	0.239	0.154 ± 0.204	0.464	0.025 ± 0.085	0.766	0.651
Diastolic	− 0.275 ± 0.079*	0.001	− 0.525 ± 0.065	< 0.001	0.168	− 0.019 ± 0.066^#^	0.774	− 0.303 ± 0.114	0.011	0.008	0.317 ± 0.087	0.001	0.199 ± 0.183	0.296	0.032
Mean	− 0.334 ± 0.074^&^	< 0.001	− 0.540 ± 0.061	< 0.001	0.081	− 0.018 ± 0.063	0.779	− 0.158 ± 0.136	0.253	0.067	0.604 ± 0.219^&^	0.016	0.147 ± 0.087	0.008	0.669

Values are regression coefficient (β) ± SE, derived from multiple linear regression analyses of coronary flow index with central aortic systolic, diastolic and mean blood pressure, respectively, after adjustment for gender, age, body mass index, current smoking, history of hypertension and dyslipidemia, prior myocardial infarction, severity of coronary artery disease, GFR, total and HDL cholesterol ratio, log-transferred hsCRP and left ventricular ejection fraction. P values for interaction in group of mild, intermediate and severe stenosis of PCDA are given

*GFR* glomerular filtration rate, *HDL* high-density lipoprotein, *hsCRP* high-sensitivity C reactive protein, *PCDA* predominant collateral donor artery

* P < 0.001, ^#^ P < 0.01 and ^&^ P < 0.05 vs. the corresponding β value in non-diabetes

## Discussion

Our results support the hypothesis that in the setting of CTO, coronary collateral flow is adversely affected by the combined effect of donor artery stenosis severity and aortic diastolic BP. Even a moderate stenosis in the PCDA resulted in lower collateral flow as diastolic BP decreases below 80 mmHg for type 2 diabetic patients compared with non-diabetic patients.

### Interactive effects of PDCA stenosis and BP on collateral flow

Physiologically, coronary collateral inflow into the distal vessel of a CTO through a variety of anatomic arteriolar connections is predominantly modulated by driving force for blood flow resulted mainly from pressure gradient across the occluded site [1, 3–5]. Central aortic pressure (especially diastolic BP) generates the distal pressure within the occluded segment, and relatively high intracoronary pressure and tangential fluid shear stress imposed on the collateral endothelium have been suggested to constitute hemodynamic stimuli for arteriogenesis [5–7] and promote collateral blood flow [14, 26, 27]. A collateral donor vessel will have a distal pressure drop with an increasing stenosis and/or lowering of diastolic BP (which contributes more to mean distal occluded pressure as diastole is longer than systole) and hence both are expected to lead to lower distal occluded pressure and CFI in the recipient CTO. In the setting of single vessel occlusion, collateral function is directly affected by systemic arterial pressure [3]. However, for patients with multi-vessel coronary disease, we would expect pressure gradient across a CTO to be reduced when the artery that supplies collateral blood flow exhibits a critical stenosis because of a pressure drop proximal to the origin of the collaterals. This may result in a further reduction in coronary collateral flow particularly when BP is decreased.

The major finding of this study is that coronary collateral flow decreased stepwise as the severity of PCDA stenosis increased, and a moderate PDCA stenosis in type 2 diabetic patients could induce similar extent of collateral flow reduction to that caused by a severe PCDA stenosis in non-diabetic patients. Interestingly, our study showed that at various degrees of PCDA stenosis, type 2 diabetic patients had lower CFI when diastolic BP decreased below 60 mmHg, and even a moderate stenotic lesion in the PCDA was associated with more reduced collateral flow as diastolic BP decreased below 80 mmHg in type 2 diabetic patients compared to non-diabetic controls. These findings highlight that the effect of PCDA stenosis on collateral flow relative to BP may be different between type 2 diabetic and non-diabetic patients. The reason for this remains unclear, but a likely explanation is the presence of more diffuse coronary atherosclerosis in a diabetic setting [28]. As shown in this study, patients with type 2 diabetes had more severe coronary artery disease as evidenced by a higher percentage of multivessel disease and a greater stenosis severity of the PCDA. It has been suggested that the presence of diffuse atherosclerotic disease in the collateral donor artery would be likely to be associated with a reduced coronary flow reserve [29], and the large increase in coronary flow through collateral donor vessels as a result of the additional flow through the collateral bed could be enough for minor atherosclerotic irregularities to generate sufficient resistance to become flow limiting [30]. Furthermore, data from prior studies which have assessed the microcirculatory function in patients with and without diabetes, have demonstrated that patients with diabetes have substantially adverse functional and structural remodeling of the coronary arterioles and even amongst those diabetic patients without known coronary artery disease, the presence of an abnormal coronary flow reserve is associated with poor outcome, comparable to non-diabetic patients with known coronary disease [31, 32]. Recently, Hinkel et al. [33] reported that diabetic human myocardial explants revealed capillary rarefaction and pericyte loss compared to non-diabetic explants. In a diabetic pig model of hibernating myocardium, hyperglycemia induced microvascular rarefaction in the myocardium even without ischemia, and capillary density further decreased in chronic ischemia hearts. This indicates that type 2 diabetes destabilized microvascular vessels of the heart and may impair the responsiveness of ischemic myocardium to pro-angiogenic factors [33]. It has been possible to determine microvascular resistance using the pressure wire technique in diabetics and non-diabetics, which could give more of an insight why diabetic patients have a lower CFI in the CTO vessel and are particularly susceptible to low diastolic BP. Previous studies have shown that there exists a pronounced increase of collateral resistance [34] and obliteration of pre-existing blood vessels in diabetes [28], suggesting that diabetic microvascular resistance is higher in the donor vessel and contributes to the obstruction of collateral flow to the CTO. All these vascular changes could contribute to a further reduction in collateral flow for patients with type 2 diabetes.

### Clinical implications

Our study findings could partly highlight the importance of an individualized BP lowering strategy [35–37] and a potential influence of stenotic lesions in the collateral donor coronary artery as a revascularization target [38–40]. In patients with multivessel disease, caution should be taken when administering anti-hypertensive therapies, as aggressive reduction in systemic BP (especially diastolic BP) may compromise collateral recruitment and exacerbate myocardial ischemia particularly for those with type 2 diabetes and stenotic lesions in the PCDA. Similarly, if the vasodilatory reserve of the arterioles in the vascular bed supplied by a chronically occluded coronary artery is completely exhausted, whereas that of the PCDA is still preserved, coronary (collateral) steal may result. This phenomenon has been reported to occur in a very high proportion of well collateralized myocardial beds [41] and is most likely to occur in patients with moderate or severe stenosis of the PCDA, as vasodilator-induced increase in flow could cause a pressure drop across the stenotic lesions, thereby lowering collateral perfusion [3, 42]. Consistent with previous findings [7], our present study also demonstrates a positive association between β blockers and CFI. The use of β blockers reduces heart rate, improves fluid shear stress at the endothelial wall, and decrease catecholamine-mediated inflammatory response, favoring coronary collateral flow.

It is now generally accepted that when presented with multivessel disease, we should aim for complete rather than incomplete revascularization [43]. PCI with drug-eluting stent implantation on severe coronary stenotic lesions has become a routine clinical practice, and clinical evidence suggests that recanalization of a CTO as a part of a complete revascularization procedure confers a substantial benefit to survival [39]. Previous studies reported that a hemodynamically ambiguous lesion would not necessarily be of low angiographic complexity, and the need to treat it might alter the long-term outcomes [30]. Our observations on the relationship between PDCA stenosis and coronary collateral flow relative to diastolic BP supports a notion that multiple aspects should be taken into consideration when planning revascularization procedures, including characteristics of totally occluded lesion, severity of PCDA stenosis, quality of collaterals, and clinical status of patients (diabetes and BP level) [44]. PCI aimed at improving collateral flow could be accomplished by reducing proximal donor artery stenosis, thereby increasing pressure at the collateral takeoff [39]. In patients with moderate PDCA stenosis (especially those with type 2 diabetes), the use of fractional flow reserve to reveal ischemia can help in clinical decision-making [40]. Furthermore, hypotension (especially low diastolic BP) should be avoided during the procedure. Whether such a strategy is particularly useful for type 2 diabetic patients with CTO and moderate PCDA stenosis warrants further investigation.

### Limitations

We acknowledge there are limitations worth mentioning. First, this study is cross-sectional for the point of coronary collateral flow investigation, thereby allowing us to detect association, not to determine the difference between diabetic and non-diabetic response of CFI to increasing diastolic pressure and to establish a causative link and to predict clinical outcome. Second, the number of patients in this study is small as compared to previous reports using angiography to assess coronary collateralization in diabetes. However, it was out-weighted by the use of a quantitative assessment of collateral function, whereas the angiographic method is a semi-quantitative grading of collateral contrast filling. Third, the use of a microcatheter to measure pressure distal to the occluded segment may be problematic as the lesion may compress the catheter and cause inaccuracy in the pressure measured. Although a pressure sensowire would have been more accurate, the absolute difference in intracoronary pressure measurement was small using a microcatheter or a pressure wire method. Finally, central venous pressure was not measured, which is not a major concern when measuring coronary fractional flow reserve but could significantly influence the CFI. Collaterals in the whole cohort were quite good (CFI > 0.25) for the majority of patients. This may be because CFI was determined on an assumption of central venous pressure. Obviously, the accuracy of CFI measurement could be significantly affected by minor variation in actual central venous pressure. Likewise, measurement of left ventricular end-diastolic pressure (LVEDP) in these patients was also important as compressive forces of the ventricle can influence collateral support via septal collaterals. It is possible that diabetic patients have stiffer left ventricle and increased LVEDP that impairs collateral support.

## Conclusions

In patients with stable coronary artery disease and CTO, donor artery stenosis confers greater risk for reduced coronary collateral flow when diastolic BP is decreased. For type 2 diabetic patients, even a moderate stenosis in the PCDA is associated with more reduced collateral flow as diastolic BP decreases below 80 mmHg compared with non-diabetic patients. These findings may provide clinical insight into the management of patients with coronary artery disease.

## Abbreviations

ANOVA: analysis of variance
BMI: body mass index
BP: blood pressure
CABG: coronary artery bypass grafting
CFI: collateral flow index
CKD-EPI: chronic kidney disease epidemiology collaboration
CTO: chronic total occlusion
CVP: central venous pressure
GFR: glomerular filtration rate
HbA1c: glycated hemoglobin
HDL: high-density lipoprotein
hsCRP: high-sensitivity C-reactive protein
LSD: least significant difference
NCEP: national cholesterol education program
NYHA: New York Heart Association
Pa: mean central aortic pressure
PCDA: predominant collateral donor artery
PCI: percutaneous coronary intervention
Pd: mean distal occluded pressure
SD: standard deviation
